# Supplementary material for: Heart function enhancement by an Nrf2-activating antioxidant in acute Y-strain Chagas disease, but not in chronic Colombian or Y-strain
Source: PLoS Negl Trop Dis. 2024 Nov 7;18(11):e0012612. doi: 10.1371/journal.pntd.0012612 (PMC11588235; doi:10.1371/journal.pntd.0012612)
Supplement: Attachment — Submitted filename: Response to Reviewers.pdf [file pntd.0012612.s007.pdf]

Dear Dr Paiva,

Thank you very much for submitting your manuscript "Heart Function Enhancement with Nrf2-Activating Antioxidant: Benefits in Acute Y-Strain Chagas Disease, Not in Chronic Colombian Strain" for consideration at PLOS Neglected Tropical Diseases. As with all papers reviewed by the journal, your manuscript was reviewed by members of the editorial board and by several independent reviewers. In light of the reviews (below this email), we would like to invite the resubmission of a significantly-revised version that takes into account the reviewers' comments.

Please implement all requirements and recommendations of Reviewers 1 to 5 and consider several reviewer comments the writing throughout the manuscript needs significant attention, in addition to line numbers. The authors are required to use a professional writing service because two reviewers have criticised the quality and clarity of writing.

We cannot make any decision about publication until we have seen the revised manuscript and your response to the reviewers' comments. Your revised manuscript is also likely to be sent to reviewers for further evaluation.

When you are ready to resubmit, please upload the following:

[1] A letter containing a detailed list of your responses to the review comments and a description of the changes you have made in the manuscript. Please note while forming your response, if your article is accepted, you may have the opportunity to make the peer review history publicly available. The record will include editor decision letters (with reviews) and your responses to reviewer comments. If eligible, we will contact you to opt in or out.

[2] Two versions of the revised manuscript: one with either highlights or tracked changes denoting where the text has been changed; the other a clean version (uploaded as the manuscript file).

Important additional instructions are given below your reviewer comments.

Please prepare and submit your revised manuscript within 60 days. If you anticipate any delay, please let us know the expected resubmission date by replying to this email. Please note that revised manuscripts received after the 60-day due date may require evaluation and peer review similar to newly submitted manuscripts.

Thank you again for your submission. We hope that our editorial process has been constructive so far, and we welcome your feedback at any time. Please don't hesitate to contact us if you have any questions or comments.

Sincerely,

Michael W Gaunt, PhD
Academic Editor
PLOS Neglected Tropical Diseases

Claudia Brodskyn
Section Editor
PLOS Neglected Tropical Diseases

****

Reviewer 5
The statistics are below professional standard. The T-test risks Type 1 error and multivariate tests are required. More generally unless parametric statistics conform to the normal distribution they cannot be used. The authors should seek to implement either the Mann-Whitney U, Wilcoxon Signed Rank or Kruskal-Wallace tests rather than large numbers of T-tests, where appropriate, and need statistical support.

We followed reviewer 5’s advice and performed Mann-Whitney U tests for most comparisons, with results similar to our previous t-tests, except concerning chronic infection with Y-strain. We recall that a previous

work analyzed *T. cruzi* infection concerning the nature of variables such as parasitemia, parasitism, https://www.nature.com/articles/s41598-017-08086-8 and found t-tests adjusted to adequate scedasticity assayed by Fisher test to be a good approach. We consulted a professional statistician who indicated ANOVA with Tukey correction (see answer below) and we found most of these tests produced results similar to Kruskal-Wallace. We corrected the Statistical Analysis, that now is read (page 9, line 366):

"Comparisons relied on unpaired Mann-Whitney U-tests (for two groups). For multiple comparisons, one-way ANOVA with Tukey correction was used. For pre-versus-post analyses, we performed Wilkoxon Signed Rank test. To determine differences in arrhythmia incidence among groups, Fisher's exact t-test was employed. A p-value below 0.05 was considered significant, and such values are highlighted next to the respective groups in figures."

Reviewer's Responses to Questions

Key Review Criteria Required for Acceptance?</br></br>
As you describe the new analyses required for acceptance, please consider the following:</br></br>

Methods</br></br>
-Are the objectives of the study clearly articulated with a clear testable hypothesis stated?</br>
-Is the study design appropriate to address the stated objectives?</br>
-Is the population clearly described and appropriate for the hypothesis being tested?</br>
-Is the sample size sufficient to ensure adequate power to address the hypothesis being tested?</br>
-Were correct statistical analysis used to support conclusions?</br>
-Are there concerns about ethical or regulatory requirements being met?</br></br>

Reviewer #1: To be more precise, add information on how many times a day the treatment doses were given. For example, was the dose of 5mg/kg administered once a day, or were there two administrations per day until reaching the dose of 5mg/kg?

We added "daily" to the treatment section in Materials and Methods (page 7, line 307). We are sorry for our mistake.

In the statistical analyses, why was the Student's t-test used for multiple comparisons? In analyses involving more than 2 groups, the use of the Student's t-test increases the risk of Type I error, as multiple t-tests do not undergo statistical correction. The more appropriate approach would be to use ANOVA, followed by post-hoc tests for multiple comparisons with correction after establishing significance. Additionally, it was not explicitly stated in the methodology, but was a normality test of the data conducted before applying a parametric test? If so, please include this information

The reviewer is right stating that Student's t-test increases the risk of Type I error. We consulted a professional statistician, who stated that:

"ANOVA allows you to reject the hypothesis that all groups have the same mean. This is done instead of pairwise comparisons to avoid the problem of multiple comparisons, which increase the chance of making a Type I error somewhere. However, the question that ANOVA answers is somewhat artificial; in real life, you are rarely interested in the conclusion "not all are equal." You want to know who is different from whom. The solution is to do pairwise comparisons with adjustments for multiple comparisons. If you are within the hypothesis where ANOVA would apply, the standard method is the Tukey-Kramer adjustment."

The Tukey-Kramer adjustment applies to normal distribution only. Concerning the variables we treated here, we performed several tests for gaussian distribution.

For instance, EKG:

Normality and Lognormality Tests

		A	B	C	D	E
		NI	_	Bz	CoPP	Bz+CoPP
1	Test for normal distribution					
2	Anderson-Darling test					
3	A2*	0.2858	0.6240	0.4063	0.6998	0.1421
4	P value	0.5704	0.0827	0.2739	0.0558	0.9552
5	Passed normality test (alpha=0.05)'	Yes	Yes	Yes	Yes	Yes
6	P value summary	ns	ns	ns	ns	ns
7						
8	D'Agostino & Pearson test					
9	K2	0.5508	2.895	2.106	5.608	0.4296
10	P value	0.7593	0.2352	0.3488	0.0606	0.8067
11	Passed normality test (alpha=0.05)'	Yes	Yes	Yes	Yes	Yes
12	P value summary	ns	ns	ns	ns	ns
13						
14	Shapiro-Wilk test					
15	W	0.9513	0.8851	0.8957	0.9078	0.9784
16	P value	0.5815	0.0688	0.2283	0.0787	0.9562
17	Passed normality test (alpha=0.05)'	Yes	Yes	Yes	Yes	Yes
18	P value summary	ns	ns	ns	ns	ns
19						
20	Kolmogorov-Smirnov test					
21	KS distance	0.1226	0.1964	0.1974	0.1973	0.1096
22	P value	>0.1000	>0.1000	>0.1000	0.0621	>0.1000
23	Passed normality test (alpha=0.05)'	Yes	Yes	Yes	Yes	Yes
24	P value summary	ns	ns	ns	ns	ns
25						
26	Number of values	14	14	9	18	10

For parasitism in cardiomyocytes, figure 1:

Normality and Lognormality Tests
Tabular results

		A	B	C	D	E	F
		-	H_2O_2	CoPP	RSV	Tempol	APO
1	Test for normal distribution						
2	Anderson-Darling test						
3	A2*	0.3339	0.5370	0.7141	1.769	0.4058	1.294
4	P value	0.4591	0.1153	0.0479	<0.0001	0.3043	0.0014
5	Passed normality test (alpha=0.05)?	Yes	Yes	No	No	Yes	No
6	P value summary	ns	ns	*	****	ns	**
7							
8	D'Agostino & Pearson test						
9	K2	3.357	4.580	4.515	18.43	4.085	21.38
10	P value	0.1866	0.1013	0.1046	<0.0001	0.1297	<0.0001
11	Passed normality test (alpha=0.05)?	Yes	Yes	Yes	No	Yes	No
12	P value summary	ns	ns	ns	****	ns	****
13							
14	Shapiro-Wilk test						
15	W	0.9292	0.8832	0.8695	0.6744	0.9151	0.7284
16	P value	0.2977	0.2022	0.0413	0.0002	0.1869	0.0007
17	Passed normality test (alpha=0.05)?	Yes	Yes	No	No	Yes	No
18	P value summary	ns	ns	*	***	ns	***
19							
20	Kolmogorov-Smirnov test						
21	KS distance	0.1360	0.2768	0.1958	0.3136	0.1264	0.2508

Here, not all groups present a perfectly normal distribution, and it remains a highly controversial question whether the same variable should be treated differently based on the results found in experimental groups. The analysis in: https://www.nature.com/articles/s41598-017-08086-8 claim for a Gaussian distribution for parasitism and parasitemia. The strategy used by authors, t-tests adjusted to adequate scedasticity assayed by Fisher's F-test, was also used by us with similar results.

Statistical analyses. All data were compared using Student's T-test adjusted to adequate scedasticity assayed by Fisher's F-test, except for organ damage, compared by Mann-Whitney-Wilcoxon's non-parametric U test. Correlations were evaluated by Bravais-Pearson's R linear correlation coefficient.

As reviewer 5 is concerned about whether these variables are indeed normally distributed and we found it is not normal for some experimental groups in some experiments, in face of all the controversy, we performed Mann-Whitney U tests for every comparison between two groups and one-way ANOVA with Tukey correction for comparisons between 3 groups or more, in order to answer the question "not all are equal". The conclusions did not change much, and we now show these comparisons instead of t-tests.

In light of these new analyses, we reconsidered the slight improvement in mechanical heart function observed during the chronic stage of Y-strain infection. This improvement was minor and now falls short of statistical significance. Therefore, we have omitted these considerations from our study.

Reviewer #2: This is an experimental study that sought to assess the impact of redox status on parasite burden in cardiomyoblasts and the effects of the Nrf2-inducer COPP on heart function in BALB/c mice infected with either DTU-II Y or DTU-I Colombian T. cruzi strains. Treatment with antioxidants CoPP,

apocynin, resveratrol, and tempol reduced parasite burden in cardiomyoblasts for both DTUI- and II-strains, while H2O2 increased it. CoPP treatment improved electrical heart function when administered during acute stage of Y-strain infection, coinciding with an overall trend towards increased survival and reduced heart parasite burden. These beneficial effects surpassed those of trypanocidal benznidazole, implying that CoPP directly affects heart physiology. CoPP treatment had beneficial impact on heart systolic function when started during chronic infection with Y-strain, an effect also achieved when performed during acute and evaluated during chronic stage. No impact of CoPP on heart parasite burden, electrical, or mechanical function was observed during the chronic stage of Colombian-strain infection, despite previous demonstrations of improvement with other antioxidants. Our findings indicate that amastigote growth is responsive to change in redox status within heart cells regardless of the DTU source, but CoPP influence on heart parasite burden in vivo and heart function is mostly confined to the acute phase.

The authors concluded that the nature of the antioxidant employed, T. cruzi DTU, and the stage of disease, emerge as crucial factors to consider in heart function studies.
This is an interesting study. I had some diifficulty following the text that copuld be improved and simplified.

We did our best to simplify the text with the help of an English specialist.

Reviewer #3: The methods used were sufficient to achieve the objectives.

Reviewer #4: The study objectives are clearly articulated, with an elegant, well-defined, testable hypothesis. All ethical and regulatory requirements were thoroughly met, with no concerns noted.

Results</br></br>
-Does the analysis presented match the analysis plan?</br>
-Are the results clearly and completely presented?</br>
-Are the figures (Tables, Images) of sufficient quality for clarity?</br></br>

Reviewer #1: The analysis matches the plan.
Results are in general clearly presented.
Figures are of good quality

Reviewer #2: (No Response)

Reviewer #3: The results achieved the initial proposed objective. However, the addition of new data would be interesting for the robustness of the manuscript.

Reviewer #4: 1. Regarding Figure 1, there is an important difference in the confluence of cells between the figures, particularly in CoPP. How did the authors explain this?

Yes, there is. The drugs change the shape, the staining and the proliferation of the cardiomyoblasts cell line. We already observed this phenomenon for macrophages and antioxidants, particularly for CoPP, in https://www.jci.org/articles/view/58525/figure/4 , as shown below. We added a sentence (page 3, line 96), "Cells changed their shape and culture confluence, particularly in the presence of Y infection and CoPP".

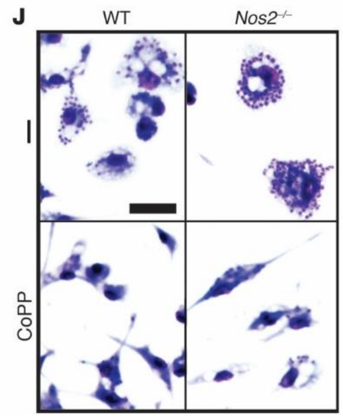

2. In Figure 2E, which section or region of the organs did the authors use? This should be clarified.

Left ventricle. We added this information to the figure (page 12, line 387). No specified portion of the liver was used.

3. In Figure 3, the authors administered a suboptimal treatment with benznidazole (25 mg/kg) and added CoPP to determine if this combination would improve heart function. Did they perform the echocardiography analysis?

No, since treatment of mice infected with Y strain does not really produce changes in the echocardiography pattern during acute phase, as can be seen in Fig 2. We did not focus on benznidazole, but on the comparison between treatment with benznidazole and CoPP.

4. In Figure 4, the author should provide a more detailed explanation of the figure, letter by letter.

We added a legend to each figure letter in order to provide a detailed explanation of the figure (page 14, lines 414 - 417):

Figure 4. **Treatment with CoPP prevents QTc prolongation and improves cardiac mechanical function at later times.** BALB/c mice were infected with 50 blood trypomastigotes of the *T.cruzi* Y strain and treated daily with CoPP (5 mg/Kg, i.p.) in the period 0-8 dpi, then assessed at the chronic period of infection, from 60-150 dpi. (A) Parasitemia (n= 6-15 mice per group), (B) Survival (n= 6-15mice per group), (C-G) EKG parameters (n= 5-12 mice per group): heart rate (RR interval); duration of PR interval; duration of P wave; duration of QRS; QT interval corrected by heart rate. (H-K) Echo parameters (n-5-9 mice per group): % Ejection Fraction (stroke volume/ end diastolic volume); Stroke volume (µL); Left Ventricle area and Right Ventricle area, mm^2, transverse section. NI = not infected; - = infected and untreated mice; CoPP = infected mice treated with CoPP.

5. In Figure 6, the authors stated that treatment with CoPP did not alter heart fibrosis, but they only assessed collagen deposition. Could the authors analyze fibronectin or other extracellular matrix proteins?

Had we accomplished an improvement in heart function in mice chronically infected with the Colombian strain and treated with CoPP, we would have certainly conducted a full study of the extracellular matrix, oxidized molecules, tissue damage, etc., to assess if it acts similarly to the antioxidant Resveratrol. In fact, we expected heart function to improve despite unchanged collagen deposition, as observed in mice chronically infected with the Colombian strain and treated with Resveratrol. In light of the failure to improve heart function, we decided to omit the fibrosis data, as it no longer serves a meaningful purpose.

Conclusions</br></br>
-Are the conclusions supported by the data presented?</br>
-Are the limitations of analysis clearly described?</br>
-Do the authors discuss how these data can be helpful to advance our understanding of the topic under study?</br>
-Is public health relevance addressed?</br></br>

Reviewer #1: Conclusions are supported by the data

Limitations are generally discussed, but the inclusion of a Limitations section on the discussion would improve the manuscript.

We added a paragraph to discussion to more clearly highlight the limitations of this study (pag 7, line 272):

We identified several limitations in our study. Although our aim was to test DTU I and II, we only used a representative strain from each. Only when the results are consistent between the two strains, it suggests a general trend across different *T. cruzi* strains, particularly regarding parasite burden in cardiomyoblasts treated with antioxidants. Furthermore, since we did not treat BALB/c chronically infected with the Y strain using resveratrol, we cannot determine whether this infection can be ameliorated by other, more suitable antioxidants. In this study, we sought to unravel variables such as *T. cruzi* source, stage of infection, and infected cell type that might contribute to the paradoxical findings reported in the literature. A more extensive, systematic investigation would be necessary to predict whether infection with a particular strain at a specific stage would benefit from treatment in terms of parasite burden or heart function, with the aim of developing broader, more universally applicable treatment strategies.

Data are discussed in the context of relevant literature.

Public health relevance is addressed. It would be important that authors discuss possile translational implications of their experimental findings, if any

The translational implications of our work are that unfortunately not every antioxidant is predicted to suit every stage/ DTU type of *T. cruzi* infection. The last sentence in discussion states: (page 7, line 289)

Though these results reinforce the safety of the use of antioxidants in CCC, they point to the need to determine which kind of antioxidant can be used in each DTU infection.

Reviewer #2: (No Response)

Reviewer #3: The conclusion of the manuscript is in accordance with the data presented.

Reviewer #4: The conclusions drawn in the study are well-supported by the comprehensive data presented.

The authors should thoroughly explore the limitations of the study.

We added a paragraph to discussion to more clearly highlight the limitations of this study (pag 7, line 272):

We identified several limitations in our study. Although our aim was to test DTU I and II, we only used a representative strain from each. Only when the results are consistent between the two strains, it suggests a general trend across different *T. cruzi* strains, particularly regarding parasite burden in cardiomyoblasts treated with antioxidants. Furthermore, since we did not treat BALB/c chronically infected with the Y strain using resveratrol, we cannot determine whether this infection can be ameliorated by other, more suitable antioxidants. In this study, we sought to unravel variables such as *T. cruzi* source, stage of infection, and infected cell type that might contribute to the paradoxical findings reported in the literature. A more extensive, systematic investigation would be necessary to predict whether infection with a particular strain at a specific stage would benefit from treatment in terms of parasite burden or heart function, with the aim of developing broader, more universally applicable treatment strategies.

Editorial and Data Presentation Modifications?</br>

Use this section for editorial suggestions as well as relatively minor modifications of existing data that would enhance clarity. If the only modifications needed are minor and/or editorial, you may wish to recommend “Minor Revision” or “Accept”.

Reviewer #1: In the introduction, could you briefly include more information about CoPP, what it is, and whether it is used in other situations, etc.

We are grateful to reviewer 1 for finding a regretful mistake in our introduction; in fact we have not mentioned the drug or its use by us in *T. cruzi* infection. In the last paragraph of introduction, we added (Page 2, lines 74-79):

In this study, we investigated the impact of antioxidants and hydrogen peroxide on cardiomyoblast parasite burden and the effects of the Nrf2-inducer CoPP (cobalt protoporphyrin) on heart function in BALB/c mice infected with either DTU II Y or DTU I Colombian *T. cruzi* strains. The antioxidant CoPP is known to act through Nrf2 activation to activate antioxidant defenses, particularly the expression of HO-1 (heme oxygenase-1) and was previously used by us in *T. cruzi* infection by Y strain in C57BL/6 mice, reducing parasitemia and macrophage parasitism [1]. Our findings confirm that, despite its positive impact on reducing parasite burden in cardiomyoblasts, antioxidant activity alone is insufficient to decrease the parasite burden in the heart or to enhance heart function during the chronic stage. The nature of the antioxidant employed, *T. cruzi* DTU, and the stage of disease, emerge as crucial factors to consider in heart function studies.

In Figure 6, wasn't it expected that the infection with the Colombian strain would cause a reduction in EF? please discuss this

Yes, it is the usual finding, a significant reduction in EF, but note that SV, which is a more direct measure of systolic function, is significantly reduced ($p=0.0018$, Mann-Whitney test, as suggested by reviewer). We recall that EF represents SV/ end diastolic volume, and reductions in diastolic function (end diastolic volume) can compensate for reductions in SV. Unfortunately, experiments take 90 days to be performed, we lose some mice, and the ethical committee does not allow large numbers of mice, so sometimes we may have a decrease in EF falling short of statistical significance.

In Figure 6, only the measurement of the RV was performed. Why wasn't the measurement of the LV done, as it was with the Y strain?

We are grateful to the reviewer for identifying this oversight. Both the RV and LV evaluations were conducted, and we have included the results in Figure 6.

Reviewer #2: (No Response)

Reviewer #3: The authors must add the bar scale in the figure 1 A and the scale value in the figure 2E.

We added the bar scale to Figs 2.

Reviewer #4: Sometimes the authors use rats, other times mice.

No, we only used BALB/c mice.

In echocardiography studies, the authors did not find a significant change in ejection fraction with the treatment. The authors should further explore and explain this result.

Yes, it is the usual finding, a significant reduction in EF, but note that SV, which is a more direct measure of systolic function, is significantly reduced ($p=0.0018$, Mann-Whitney test, as suggested by reviewer). We recall that EF represents SV/ end diastolic volume, and reductions in diastolic function (end diastolic volume) can compensate for reductions in SV. Unfortunately, experiments take 90 days to be performed, we lose some mice, and the ethical committee does not allow large numbers of mice, so sometimes we may have a decrease falling short of statistical significance.

Summary and General Comments</br></br>

Use this section to provide overall comments, discuss strengths/weaknesses of the study, novelty, significance, general execution and scholarship. You may also include additional comments for the author, including concerns about dual publication, research ethics, or publication ethics. If requesting major revision, please articulate the new experiments that are needed.

Reviewer #1: This is an original work that tests the hypothesis that the use of an Nfr2-inducer (CoPP) during acute or chronic infection could have beneficial effects on mouse infected with different strains of T. cruzi. The research is based on literature data suggesting that oxidative stress is important for the growth of the parasite. Among the findings, the beneficial effects of using CoPP seem to be present in the acute phase, without showing significant differences in the chronic phase of the disease. The work is well-constructed, supported by literature data, and includes previous work from the group.

Reviewer #2: (No Response)

Reviewer #3: It is an exciting manuscript showing the antioxidants benefits in acute Y Strain infection, focusing on heart function enhancement. The article presents an interesting idea concerning the Nrf2-HO1 axis in Y-strain infection. However, some points can be better discussed.

Major points:

1) Although Shan and colleagues (2006) as well as other articles have shown that the effect of CoPP on HO-1 induction can be dependent on Nrf2, there is a robust literature showing that CoPP can be an inducer of HO-1. To elucidate this issue, it would be interesting for the authors to show the activation of Nrf2 (e.g. luciferase assay), and Nrf2 and HO1 expression (e.g. western blotting). The presentation of the Nrf2-HO-1 axis would make the manuscript more robust.

The reviewer is right, but we refer to our previous work showing that this is indeed the case, even during *T. cruzi* infection https://www.ncbi.nlm.nih.gov/pmc/articles/PMC3386808/: in Fig 3E we show that CoPP acts through Nrf2 to reduce parasitism in macrophages. In Suppl Fig2B we show that CoPP induces HO-1 expression even on infected cells, as shown below. We added a sentence to first appearance of CoPP in results to properly refer to our previous publication as a source of this information (page 3, line 103).

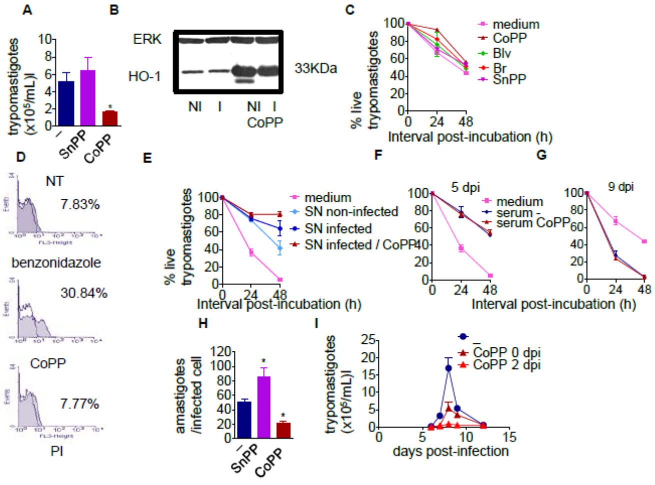

Supplemental Figure 2. CoPP does not directly kill trypomastigotes, interfere with intracellular amastigogenesis or induce secretion of a soluble killing factor. **(A)** Effects of treatment with CoPP and SnPP on the mean trypomastigote number in thioglycollate-elicited macrophages. Trypomastigotes were counted in supernatants after 5 days of cell culture. **(B)** Effects of CoPP and SnPP on the expression of HO-1, as shown by immunoblot. **(C)** Effects of treatment with CoPP, SnPP, biliverdin (Blv), or bilirubin (Bb) on mean trypomastigote survival, as assessed by motility. Three independent wells were counted for each point. **(D)** Effects of treatment with CoPP or benzonidazole (100µM) on the viability of trypomastigotes, assessed by iodide propidium (IP) exclusion (flow cytometry). **(E)** Effects of treatment with supernatants (SN) from derived macrophages as in (A) on trypomastigote survival. Three independent wells were counted for each point. **(F)** Effects of treatment with serum derived from mice treated in vivo with CoPP, SnPP or left untreated, at 5 dpi or **(G)** 9 dpi. **(H)** Effects of treatment with CoPP and SnPP on the mean amastigote number in thioglycollate-elicited macrophages; CoPP was present from 24h after infection on (post-amastigogenesis period). **(I)** Effects of treatment in vivo with CoPP starting 2 days after infection on the mean parasitemia (n=8). Controls treated with CoPP starting 2h after infection (usual regimen) are also shown. NI= non-infected; -=infected non-treated controls. Errors bars represent SEM. All experiments were repeated at least twice with similar results, except for F.

2) A comparison with the classical Nrf2 activator (DMF or MMF) and the classical HO-1 activator (heme, in the appropriate concentration) would be interesting.

We agree with reviewer 3. In our previous publication https://www.ncbi.nlm.nih.gov/pmc/articles/PMC3386808/ we used Nrf2-activators that have known off-target effects, such as resveratrol, pterostilbene, oltipraz; we also used HO-1 inhibitor SnPP to account for the effects of HO-1 activity and the genetic manipulation of Nrf2 / HO-1, to account for direct Nrf2 and HO-1 effects. Therefore, here we used our previous knowledge of CoPP as a Nrf2 activator in *T. cruzi* infection with just some occasional controls to assess its effects.

Though we are more focused on testing whether the effects of antioxidants in general in reducing parasitism can be extended to cardiomyoblasts, here we show a classical Nrf2-activator, tBHQ, and its effects on parasite burden of cardiomyoblasts H9C2 infected with Colombian strain. The classical Nrf2-activator was capable of reducing parasite burden similar to CoPP and all other antioxidants tested (resveratrol, apocynin, tempol).

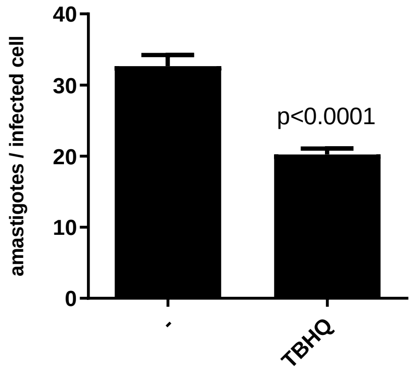

We recall that tBHQ is a classical Nrf2 activator that works in cardiomyoblasts H9C2 https://pubmed.ncbi.nlm.nih.gov/27220726/ to preserve their viability upon oxidative stress. A more

systematic work would be required to ascertain whether the effects of classical Nrf2 activators are similar to that of CoPP in vivo.

3) The authors stated in the manuscript that they changed the redox status when they treated the cells with H2O2. However, the statement is very weak. To evaluate the redox status, authors should evaluate the production of reactive oxygen species (e.g. DCF, CellRox...) and/or antioxidant activity and/or oxidative damage (shown only in figure 6). I encourage the authors to evaluate the redox balance, as it would be interesting to understand how the non-damaging oxidative burst occurs, and which reactive oxygen species are involved.

We agree with the reviewer that our statement regarding the change in redox status needs to be supported by direct measurements of redox signaling pathways and oxidative damage. Nevertheless, it is a common assumption that H_2O_2 alters the redox status https://pubmed.ncbi.nlm.nih.gov/25671543/ and we resort to our previous work in which all these measures were performed https://pubmed.ncbi.nlm.nih.gov/22728935/.

To strengthen our analysis, we refer to the study by De Angelis et al. (2012), which measured ROS production in H9C2 cardiomyoblasts treated with H_2O_2 and demonstrated significant oxidative stress and redox imbalance https://jbiomedsci.biomedcentral.com/articles/10.1186/1423-0127-21-56 . This study provides evidence supporting our assumption that H_2O_2 treatment changes the redox status by increasing ROS levels and oxidative stress. Moreover, previous studies, such as the investigation of oxidative lesions in *Trypanosoma cruzi* amastigotes treated with H_2O_2 https://www.ncbi.nlm.nih.gov/pmc/articles/PMC3681716/pdf/pntd.0002279.pdf, also support our initial assumption.

Therefore, we modified our statement to "the oxidative environment" instead of “redox status” and acknowledged the existing literature that has measured ROS production in similar experimental setups (page 1, lines 16, 27; page 5 – line 209).

2) The use of H_2O_2 is very superficial, because the production of reactive oxygen species can occur by different sources and in different compartments, therefore it would be interesting to use selective NOX inducers.

In another study, we demonstrated that pro-oxidants other than H_2O_2, such as paraquat, can also favor an increased parasite burden in macrophages https://pubmed.ncbi.nlm.nih.gov/22728935/. The use of H_2O_2 itself has been widely recognized in *T. cruzi* infection research through various studies, both in the context of mammalian cell infection https://www.ncbi.nlm.nih.gov/pmc/articles/PMC3681716/pdf/pntd.0002279.pdf, https://pubmed.ncbi.nlm.nih.gov/22728935/, https://pubmed.ncbi.nlm.nih.gov/27035573/, and the differentiation and proliferation of epimastigotes and amastigotes https://pubmed.ncbi.nlm.nih.gov/32861766/, https://pubmed.ncbi.nlm.nih.gov/25671543/, https://www.ncbi.nlm.nih.gov/pmc/articles/PMC3191175/.

The purpose of using H_2O_2 in this study is not to pinpoint the exact source of reactive oxygen species production, which indeed warrants a comprehensive and systematic exploration. Instead, our objective is to offer a counterpoint to the use of antioxidants. This approach is intended to demonstrate that the oxidative environment, influenced by the presence of H_2O_2, significantly dictates the fate of parasitism. We believe this perspective adds valuable insights into the understanding of oxidative stress and its impact on parasitic infections.

We added the sentence and the reference on the effects of H_2O_2 to page 2 line 95: Treatment with H_2O_2 produces oxidative damage and activates redox signaling pathways [17].

Minor points:

1) It would be interesting to discuss that SnPP also inhibits HO-2. ZnPP is more selective for HO-1.

We added the sentence and the reference to the text (page 3 - line 109): “Mice treated with SnPP (an inhibitor of heme oxygenase activity [18])”

2) It was not clear why the authors only treated the cells with Colombian strain chronically. Although there were promising results in figure 1, it was only in the last figure that the authors returned to presenting the data involving the Colombian strain.

This work was performed to fulfill some gaps in the literature, as previously pointed out by us in a review https://journals.plos.org/plospathogens/article?id=10.1371/journal.ppat.1006928 .

Fig 1 was intended to offer a comparison between Colombian (DTU I) and Y strain (DTU II) that could solve the question posed by a previous publication concerned the effects of antioxidants on infected cardiomyocytes and described in introduction: “However, while the antioxidant enzyme catalase decreased the parasite burden in cardiomyocytes infected with *T. cruzi* JG (D,TU II), it did not alter the burden of cardiomyocytes infected with Col1.7G2 (DTU I) [8].”

Thus, the effect on cardiomyocyte infection exists and is independent of the *T. cruzi* strain. However in vivo we expected that not only parasite burden depends on factors other than direct effects of CoPP on cardiomyocytes, but also heart function depends on factors other than parasite burden.

Concerning heart function of mice acutely infected with Y-strain, we intended to fulfill the gap led by our previous study https://pubmed.ncbi.nlm.nih.gov/22728935/ , in which we showed decreased heart parasitism in C57BL6 mice treated with CoPP, but did not extend the study to heart function. Here we show in mice more susceptible to heart disease, BALB/c, that CoPP ameliorates heart function in mice acutely infected with Y-strain, an effect that has two components, reduction in parasite burden and direct effects on heart physiology.

We then wanted to determine whether this effect of CoPP on heart function of mice acutely infected with Y strain could be extended to the chronic stage. We found that it could be detected during chronic stage only when treatment was performed during acute stage, most likely because of reduced parasite burden. As this data contradict the improvement in heart function produced by treatment with other antioxidants (resveratrol, tempol) during chronic stage of Colombian-strain infection, we treated mice chronically infected with Colombian strain with CoPP. When treated during chronic stage, mice infected with Colombian strain do not benefit from CoPP just like mice infected with Y strain, allowing us to conclude that despite its direct effects on heart function during acute stage of Y-strain infection, CoPP is not appropriate to the chronic stage.

Note that despite the direct effects on parasite burden in cardiomyocytes and the benefits to heart function of mice acutely infected with Y strain, CoPP does not seem to be appropriate to the main problem in Chagas disease, which is the heart dysfunction diagnosed during chronic stage, different from resveratrol https://pubmed.ncbi.nlm.nih.gov/27788262/ . It remains to be solved whether treatment with resveratrol succeeds to improve heart function of mice infected by other *T. cruzi* DTUs.

3) The authors should review some typos.

We did our best using gpt to correct the typos.

4) The authors must add the bar scale in the figure 1A and the scale value in the figure 2E. We added the bar scales to the figures 1 and 2E.

Reviewer #4: This study represents an important effort in exploring the influence of redox status on parasite burden in cardiomyoblasts, as well as evaluating the impact of the Nrf2-inducer COPP on heart function in BALB/c mice infected with either DTU-II Y or DTU-I Colombian T. cruzi strains. The novelty of these findings contributes significantly to the current literature on Nrf2-activating antioxidants. The study's importance is highlighted by its potential implications for advancing our understanding of Chaga disease physiopathology.

PLOS authors have the option to publish the peer review history of their article (what does this mean?). If published, this will include your full peer review and any attached files.

If you choose “no”, your identity will remain anonymous but your review may still be made public.

Do you want your identity to be public for this peer review? For information about this choice, including consent withdrawal, please see our Privacy Policy.

Reviewer #1: No

Reviewer #2: No

Reviewer #3: Yes: João Alfredo de Moraes

Reviewer #4: No

Figure Files:
While revising your submission, please upload your figure files to the Preflight Analysis and Conversion Engine (PACE) digital diagnostic tool, https://pacev2.apexcovantage.com. PACE helps ensure that figures meet PLOS requirements. To use PACE, you must first register as a user. Then, login and navigate to the UPLOAD tab, where you will find detailed instructions on how to use the tool. If you encounter any issues or have any questions when using PACE, please email us at figures@plos.org.

Data Requirements:

Please note that, as a condition of publication, PLOS' data policy requires that you make available all data used to draw the conclusions outlined in your manuscript. Data must be deposited in an appropriate repository, included within the body of the manuscript, or uploaded as supporting information. This includes all numerical values that were used to generate graphs, histograms etc.. For an example see here: http://www.plosbiology.org/article/info%3Adoi%2F10.1371%2Fjournal.pbio.1001908#s5.

Reproducibility:
To enhance the reproducibility of your results, we recommend that you deposit your laboratory protocols in protocols.io, where a protocol can be assigned its own identifier (DOI) such that it can be cited independently in the future. Additionally, PLOS ONE offers an option to publish peer-reviewed clinical study protocols. Read more information on sharing protocols at https://plos.org/protocols?utm_medium=editorial-email&utm_source=authorletters&utm_campaign=protocols
